# Randomized Prospective Comparison of the Singularity^TM^ Air Laryngeal Mask and Two Second-Generation Laryngeal Masks in Adult Patients

**DOI:** 10.3390/jcm14238513

**Published:** 2025-11-30

**Authors:** Danijel Novina, Nadja Ettlin, Norbert Nickel, Norbert Aeppli, JoEllen Welter, Alexander Dullenkopf

**Affiliations:** Institute for Anesthesiology, Spital Thurgau Frauenfeld, 8501 Frauenfeld, Switzerland; danijel.novina@stgag.ch (D.N.); nadja.ettlin@stgag.ch (N.E.); norbert.nickel@stgag.ch (N.N.); norbert.aeppli@stgag.ch (N.A.); joellen.welter@stgag.ch (J.W.)

**Keywords:** laryngeal mask, airway management, general anesthesia

## Abstract

**Background/Objectives:** Supraglottic airway devices are commonly used airway management tools, with various second-generation laryngeal masks available for patients undergoing general anesthesia. These devices offer improved sealing, gastric suction capabilities, and the potential for tracheal intubation. This study compared the recently introduced Singularity^TM^ Air laryngeal mask with two well-established devices, Ambu^®^ AuraGain^TM^ and i-GEL^®^, under clinical conditions. **Methods:** We prospectively included 98 adult patients scheduled for elective surgery requiring general anesthesia. Patients were randomized to one of three laryngeal mask groups, and data on insertion success, ventilation efficiency, and postoperative complications, such as sore throat and dysesthesia, were collected. The primary endpoint was oropharyngeal sealing pressure, with additional assessments of insertion ease and bronchoscopic glottic visibility. **Results:** Median initial oropharyngeal leak pressure was lowest with i-GEL^®^ (22 cm H_2_O) as opposed to Ambu^®^ AuraGain^TM^ (25 cm H_2_O) and Singularity^TM^ Air (25 cm H_2_O) [*p* = 0.0138], but this difference equalized after 15–30 min. I-GEL^®^ showed higher insertion success (88%, *p* = 0.001) and shorter time-to-first ventilation (29 s, *p* = 0.0106). Conversely, the gastric tube insertion rate was lower (70% versus >90% in the other masks). The Ambu^®^ AuraGain^TM^ and Singularity^TM^ Air performed similarly for most parameters. No significant differences were observed in tracheal intubation potential or postoperative adverse events among the three groups. **Conclusions:** The Singularity^TM^ Air performed comparably to Ambu^®^ AuraGain^TM^ and i-Gel laryngeal masks in oropharyngeal sealing pressure. I-Gel^®^ had the highest successful insertion rate. Most of the differences detected were not statistically significant, with all three masks providing effective airway management.

## 1. Introduction

Supraglottic airway devices (SGAs) are widely used airway management tools for patients undergoing general anesthesia [1]. These devices were first introduced in the 1980s as a less invasive alternative to endotracheal intubation, still considered the gold standard for some indications (e.g., mechanical ventilation with requirement of high inspiratory or end-expiratory pressure, risk for pulmonary aspiration) [2,3]. The primary advantages of SGAs are that they require less technical expertise (easier and faster to insert), generally do not require neuromuscular blocking agents for insertion, which results in less pulmonary post-operative complications, and result in less airway trauma and complications [1,4]. Consequently, a variety of SGAs have since been developed and are commercially available worldwide, with the laryngeal mask (LM) being the most commonly used [1,5]. These masks are not without shortcomings, though. Among those are inadequate seal, leakage, displacement, and risk of obstruction [5,6].

Second-generation laryngeal masks were designed to overcome some of the limitations of the initial laryngeal masks [7]. These newer laryngeal masks provide reliable sealing pressure, incorporate a gastric suction mechanism, and, when necessary, allow for tracheal intubation through the laryngeal mask. The recently introduced Singularity Air^TM^ laryngeal mask (Singularity AG, Maur/Zurich, Switzerland) has the additional benefit of a dial to bend or adjust the device’s shaft angle after insertion [8]. This design feature is believed to support better sealing performance. To date, evaluations of the Singularity Air^TM^ have been carried out in controlled laboratory settings or as observational, non-controlled studies [8,9].

This study’s aim was to compare the Singularity Air^TM^ laryngeal mask with two well-studied second-generation laryngeal masks (Ambu^®^ AuraGain^TM^ (Ambu, Bad Nauheim, Germany) and i-GEL^®^ (Intersurgical, Sankt Augustin, Germany)) under clinical conditions with adult patients. The primary endpoint was oropharyngeal sealing pressure.

## 2. Materials and Methods

### 2.1. Setting and Participants

This comparative study was conducted from July 2023 to May 2024 at a Swiss acute care hospital performing approximately 9000 anesthetics per year. Ethical approval was obtained from the Ethics Committee of Eastern Switzerland (EKOS 21/191, 09 November 2021). The study was registered in the German Clinical Trials Register (DRKS00029113, 24 May 2022) and carried out according to the Declaration of Helsinki. Reporting followed CONSORT (CONsolidated Standards Of Reporting Trials) guidelines. Consecutively treated patients over the above-mentioned period were considered for inclusion. All patients gave written informed consent before being enrolled in the study.

We prospectively assessed for inclusion all adult (18 years and older) patients eligible for airway management with a laryngeal mask who were undergoing elective surgery under general anesthesia according to institutional standards. Inclusion required an American Society of Anesthesiologists (ASA) physical status of I to III and having fasted in accordance with internal guidelines (i.e., 6 h without solid food, clear liquid allowed up to time of anesthesia). The setting was real-world clinical practice with resource constraints for research, which prolonged the inclusion period.

The main exclusion criterion was any condition that contraindicated airway management with laryngeal mask at our institution, such as morbid obesity (body mass index (BMI) at or above 35 kg/m^2^), symptomatic esophageal reflux disease, surgery in lateral or prone position, prolonged Trendelenburg position, laparoscopic surgery, or ear-nose-throat surgery. Additionally, we excluded patients who were pregnant, undergoing emergency treatment, had loose teeth, faced challenges in providing informed consent (e.g., language problems, psychological disorders, dementia), who were participating in another study, and who had previously participated in this current investigation.

Using a pre-generated randomized number list, study participants were assigned to one of three laryngeal mask brands. Given that simple randomization without blocking or stratification was used, some imbalance in group sizes occurred by chance.

### 2.2. Study Devices

**Figure 1:**
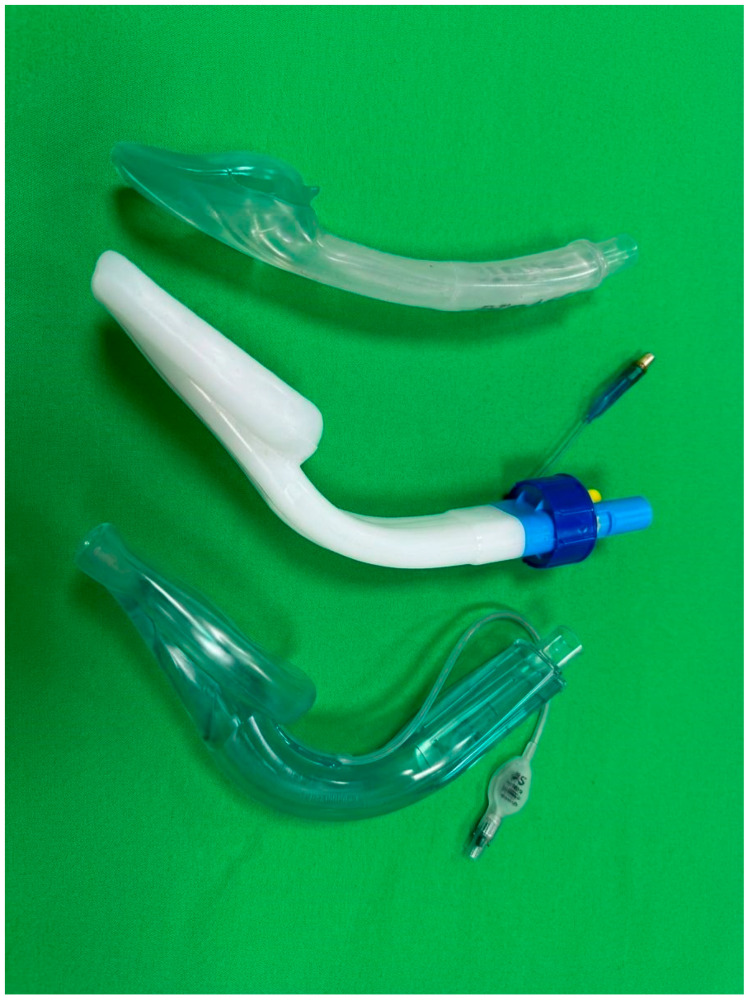
Tested laryngeal masks. From top: i-GEL^®^, Singularity^TM^ Air, Ambu^®^ AuraGain^TM^.

The three laryngeal mask brands were: (I) Singularity^TM^ Air, (Singularity AG, Maur/Zurich, Switzerland), (II) Ambu^®^ AuraGain^TM^ (Ambu, Bad Nauheim, Germany), (III) i-GEL^®^ (Intersurgical, Sankt Augustin, Germany) (Figure 1).

Device size was based on manufacturer recommendations. For the Singularity^TM^ Air laryngeal mask, patients ≤ 70 kg received size 4, whereas those >70 kg received size 5. The Ambu^®^ AuraGain^TM^ laryngeal mask is available in three different adult sizes. For this study, size 4 was selected for patients up to 90 kg and size 5 for heavier patients. The i-GEL^®^ laryngeal mask, which comes in four adult sizes, was applied in size 4 for individuals up to 70 kg and in size 5 for patients >70 kg.

In preparation for use, the cuffs of the Singularity Air^TM^ and AuraGain^TM^ laryngeal masks were nearly emptied using a syringe, and a sterile lubricant was applied to both the outer and inner curvatures of each device (Instillagel; FARCO-PHARMA, Köln, Germany). The Singularity Air^TM^ required an additional step in which the shaft was slightly bent as per the manufacturer’s instructions.

### 2.3. Anesthesia Procedures

Anesthesia was administered in accordance with our institution’s standards. Thirty minutes before transfer to the operating room, patients received 3.75–7.5 mg. of oral midazolam. Routine monitoring was applied (non-invasive blood pressure, electrocardiogram, pulse oximetry; IntelliVue MP30; Philips, Zurich, Switzerland), and a peripheral venous catheter was inserted. Depth of anesthesia was assessed with an EEG-based Bispectral Index (BIS) monitor (Philips, Zurich, Switzerland).

Prior to induction, patients continued spontaneous breathing while receiving oxygen until the end-tidal FiO_2_ reached 0.7. A propofol-based anesthetic was supplemented with remifentanil (target-controlled infusion (TCI), Schnider pharmacokinetic model for propofol, Minto pharmacokinetic model for remifentanil (Alaris PK Syringe Pump, CareFusion, Rolle, Switzerland)). Induction targets were set at 6 mcg/mL for propofol and 2 ng/mL for remifentanil. Additionally, 1–3 mcg/kg of fentanyl was administered intravenously. Insertion of the laryngeal mask was performed once the BIS value had fallen below 60 and bag-mask ventilation was smooth. After securing the airway, propofol and remifentanil were reduced to maintain a BIS value between 40 and 60. Fentanyl was supplemented, and additional medications, such as basic analgesia or PONV prophylaxis, were provided at the discretion of the responsible anesthesia personnel.

The responsible anesthesiologist determined the exact time point for laryngeal mask insertion. A second member of the anesthesiology team positioned the patient’s head and opened the patient’s mouth, performing an Esmarch maneuver. If initial attempt at insertion was unsuccessful, the following approaches were utilized: deepening the level of anesthesia, applying an alternative insertion approach (e.g., 180° rotation, increased pre-bending of the Singularity Air^TM^ shaft, or digital guidance), or administering a neuromuscular blocking drug. Once inserted, the Singularity Air^TM^ or AuraGain^®^ cuffs were inflated with a hand-operated manometer (Cuff manometer; VBM, Sulz am Neckar, Germany) to a cuff pressure of 60 cm H_2_O. If manual bag ventilation was insufficient after insertion, further steps included deepening anesthesia, repositioning the device, adjusting the curvature of the Singularity Air^TM^, or administering neuromuscular blockade. Persistent difficulty with insertion or ventilation led to the use of an alternative airway device, typically a first-generation laryngeal mask or endotracheal intubation, in line with institutional standards.

During anesthesia, patients were ventilated mechanically using either Atlan^TM^ or Primus^TM^ ventilator (Dräger, Lübeck, Germany), with tidal volume, respiratory rate, and positive end-expiratory pressure (PEEP) set according to institutional practice. When mechanical ventilation was insufficient (inadequate tidal volume or unreliable CO_2_-tracking), ventilator parameters were adapted (PEEP reduced, inspiratory time increased, breathing frequency reduced), or the laryngeal mask was adjusted. A lubricated gastric tube (14 Ch; P.J. Dahlhausen & Co. GmbH, Köln, Germany; Instillagel; FARCO-PHARMA, Köln, Germany) was then introduced through the device’s gastric channel. Due to the smaller diameter of the gastric lumen in the i-GEL^®^ laryngeal mask, a 10 Ch gastric tube was used instead. Successful gastric placement was confirmed by auscultation of insufflated air.

The position of the laryngeal masks was assessed using endoscopy. The laryngeal mask was disconnected from the respirator, and a lubricated fiber-optic bronchoscope moved from the laryngeal mask’s shaft to its cuff. At that point, the position of the laryngeal mask and the possibility of advancing the bronchoscope into the trachea were assessed. The laryngeal mask was then reconnected to the respirator. Further anesthetics and emergence from general anesthesia were performed according to institutional guidelines.

### 2.4. Variables and Data Measurements

Oropharyngeal leak pressure (study’s primary endpoint) was assessed by increasing the airway pressure until air began to audibly escape from the patient’s mouth. The ventilator bag was switched to manual mode, the adaptive pressure release valve (APV) of the circle circuit was set to 40 cm H_2_O, and fresh gas flow was maintained at 8 L/min. Airway pressure was then raised by manually compressing the reservoir bag, up to a limit of 25 cm H_2_O. This procedure was performed after securing the airway and then repeated after 15 to 30 min.

We classified laryngeal mask insertions and manual or mechanical ventilation as “no need for intervention” if none of the above-mentioned measures for improving insertion or ventilation were necessary. Furthermore, we measured the time from discontinuation of bag-mask ventilation to the first effective manual breath through the device. “Successful ventilation” required both chest movement and a stable capnography signal. Oxygen saturation drops below 90% were documented. Likewise, gastric tube insertion was classified as “successful without intervention” or “problematic/not successful”.

With regard to the positioning of the laryngeal masks, we used the scoring system proposed by Brimacombe and Berry, which ranges from zero (failure to function, cords not seen) to four (only cords seen) [10]. The responsible anesthetist graded the ability to advance the bronchoscope into the trachea on a scale from zero (not possible; glottic opening not visible) to four (very easy; clear sight).

As patients awakened from anesthesia, the laryngeal mask was removed and its distal end checked for visible blood. The total duration of anesthesia was noted from the anesthesia record. On postoperative day one, patients were contacted in person or by telephone if outpatient surgery was performed. They were asked to rate throat discomfort from zero (no discomfort at all) to ten (worst soreness imaginable) numerical scale. Furthermore, they were asked about swallowing difficulties or abnormal sensations in the mouth or pharynx and whether any treatment had been required. Patients were instructed to notify the study team if symptoms persisted.

### 2.5. Sample Size Consideration

No a priori sample size calculation was performed. This study was designed as an exploratory comparison of three second-generation laryngeal masks, and the final sample size was determined by feasibility during the predefined study period and the number of eligible patients meeting inclusion criteria.

### 2.6. Statistical Methods

Continuous variables were presented as either mean or standard deviation or median and interquartile range based on Shapiro–Wilk test for normality. Categorical data were reported as frequency and proportions. Three-group comparisons were done using one of the following statistical tests: one-way ANOVA, Fisher’s exact test, and Kruskal–Wallis test. A Bonferroni adjustment was applied to account for the three pairwise comparisons. As such, the significance level was set at 0.0167. Post hoc tests for parameters that were found to be statistically significant were performed using Fisher’s exact test or Mann–Whitney U-test. All analyses were conducted in Stata (version 15, StataCorp, College Station, TX, USA).

## 3. Results

**Figure 2:**
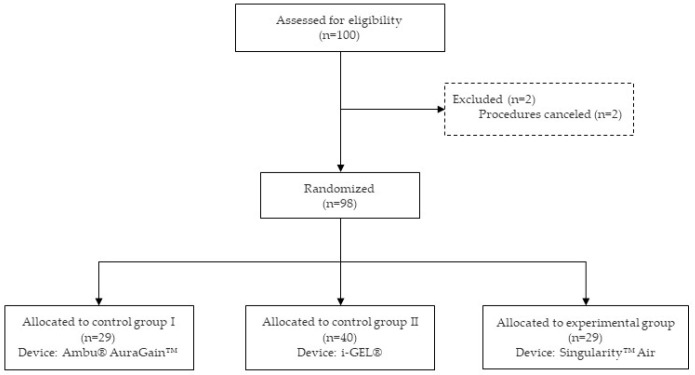
Flow diagram of the progress through the phases of randomized trial of three groups.

Of the 100 consecutively enrolled patients, two operations were canceled before anesthesia induction (Figure 2). Consequently, 98 patients were included in the final analysis (29 Singularity^TM^ Air, 40 i-GEL^®^, and 29 Ambu^®^ AuraGain^TM^). In terms of patient demographics and anesthesia-related data, no statistically significant differences among the study groups were observed (Table 1).

Regarding the oropharyngeal leak pressure, the median values initially differed among the three groups (*p* = 0.0138). Post hoc analyses showed that the lowest median pressure was in patients with the i-GEL^®^ laryngeal mask (22 cm H_2_O (IQR 15–25)). This median value was statistically significantly different from the Ambu^®^ AuraGain^TM^ (25 cm H_2_O (IQR 25–25), *p* = 0.0014) yet not the Singularity^TM^ Air (25 cm H_2_O (IQR 21–25), *p* = 0.0489). After 15 to 30 min, the oropharyngeal leak pressure increased to a median of 25 cm H_2_O in all three groups (Table 2), and no statistically significant difference was detected.

Insertion of the laryngeal mask was performed without problems in 65% of the study patients. However, the rate was significantly better with the i-GEL^®^ laryngeal mask (88%) when compared to the Ambu^®^ AuraGain^TM^ (52%, *p* = 0.004) and the Singularity^TM^ Air (48%, *p* = 0.001). However, the comparison between the latter two masks did not show a significant difference (*p* = 0.793). The median time-to-first ventilation was 33 s (IQR 26–46) for the entire cohort, and shortest duration occurred with the i-GEL^®^ laryngeal mask (29 s (IQR 25–42), which was significantly faster than the Ambu^®^ AuraGain^TM^ (39 s (IQR 32–52) *p* = 0.0033) but not the Singularity^TM^ Air (34 s (IQR 29–43) *p* = 0.1049). No significant difference in the time-to-first ventilation was observed between the Ambu^®^ AuraGain^TM^ and the Singularity^TM^ Air devices (*p* = 0.1611) [Table 2]. A peripheral oxygen saturation of <90% did not occur in any of the patients.

Manual bag-mask ventilation was successful (achieved without intervention) in 92% of the patients, and no differences were observed among the groups (*p* = 0.241). Mechanical ventilation was successful without intervention in 85% of the patients, again with similar results (*p* = 0.807) among the groups (Table 2). In four patients (n = 1 Ambu^®^ AuraGain^TM^, n = 3 i-GEL^®^ laryngeal masks; *p* = 0.382), mechanical ventilation was unsuccessful, necessitating the use of an alternative airway device.

Insertion of the gastric tube was successful without intervention in 84%, and did not differ significantly among the laryngeal mask types (*p* = 0.058). However, in the i-GEL^®^ group, the rate was notably lower (70%) than the other two mask groups (>90%). Additionally, the i-GEL^®^ required the use of a smaller gastric tube, and placement was unsuccessful in three patients, compared with one failure in the Ambu^®^ AuraGain^TM^ group.

Regarding potential intubation through the laryngeal mask, the grading of the view to the glottis opening in the entire cohort was 4 (IQR 3–4) on a scale from 0 to 4 (4 best score), and no differences were detected among the groups. Likewise, the anesthetist’s grading of potential intubation success was a median of 4 (IQR 3–4).

Blood staining on the device tip after removal occurred in 2% of cases (n = 1 Ambu^®^ AuraGain^TM^, n = 1 Singularity^TM^ Air laryngeal masks). Although no persisting complications occurred, one patient developed a hematoma at the base of the tongue (Ambu^®^ AuraGain^TM^ group). The median score for measuring soreness of throat in the entire cohort was zero (IQR 0–0), with no difference among the mask types. Five percent of the patients reported difficulties in swallowing. Although the highest rate was 10% for those patients who had a Singularity^TM^ Air mask, distributions among the mask types were not statistically significant (*p* = 0.438). Lastly, the rate of hypo-/dysesthesia was 5% for the cohort, which was similarly distributed among the groups (*p* = 0.859). The highest rate reported was 7% in the Singularity^TM^ Air group.

## 4. Discussion

For this study, we compared two well-tested second-generation laryngeal masks to the recently released Singularity^TM^ Air. In terms of our primary endpoint, the test laryngeal mask performed similarly to the Ambu^®^ AuraGain^TM^. Although the i-GEL^®^ masks resulted in a lower initial median oropharyngeal leak pressure, these findings were not significantly different from the Singularity^TM^ Air. Comparisons across most indicators revealed that the Singularity^TM^ Air most resembled the Ambu^®^ AuraGain^TM^. The i-GEL^®^ masks outperformed the other two laryngeal masks in a few key outcomes, such as a faster time-to-ventilation and a higher laryngeal mask insertion rate.

Laryngeal masks are commonly used in anesthesia, with the i-GEL^®^ and Ambu^®^ AuraGain^TM^ being extensively studied [1,5,7,11,12,13,14]. In a recent meta-analysis, the i-GEL^®^ served as a reference comparator for patient-centered outcomes [7]. Laryngeal masks are recognized for their reliability and ease of use [1]. The findings of this study further support these advantages, demonstrating an overall ventilation success rate of 97% during anesthesia. Likewise, the median time from the start of laryngeal mask insertion to the first successful mechanical ventilation was 33 s, which clinically is comparable to other studies and meta-analyses [7,11,15,16]. There are studies reporting quicker insertion times for laryngeal masks. Unfortunately there is no real standard for assessing the time to successful ventilation when testing laryngeal masks. We preferred a real-time scenario with stopping bag mask ventilation, storing the bag, positioning the head, opening the patient’s mouth, gently inserting the laryngeal mask, blocking it, carefully re-connecting the bag after removing gloves etc., without pushing participants for quick insertion. There was no patient experiencing a peripheral oxygen saturation of 90% or lower. Adequate placement and oropharyngeal sealing were the main contributors to these high success rates.

In this study, the oropharyngeal leak pressure was tested up to a maximum of 25 cm H_2_O. This limit was selected because with the chosen method of testing the increase in airway pressure generates positive continuous airway pressure (CPAP) that cannot be held or increased excessively. All three laryngeal masks sealed the airway up to this limit after approximately 20 min—a time most likely needed for warming or softening of the laryngeal mask and improved adaption to the pharynx.

Insertion was easiest in the i-GEL^®^ group, with nearly 90% of cases considered problem-free. In contrast, the other two laryngeal masks more often required basic techniques, such as finger-assisted guidance or inverse insertion, to facilitate insertion. This may be due to the i-GEL^®^ laryngeal mask’s non-inflatable cuff, making it less bulky than the other two, and is in accordance with other studies [7,17,18,19]. Since most anesthesia personnel are accustomed to overcoming insertion challenges, successful ventilation was achieved within an appropriate timeframe.

Another advantage of the second-generation laryngeal masks is the possibility of inserting a gastric tube for draining fluid or air from the stomach. The overall success rate for this study was 84%, with the lowest success rate (70%) in the i-GEL^®^ laryngeal mask. This may be attributed to the smaller diameter of the gastric tube channel in the i-GEL^®^ compared to the Singularity^TM^ Air and Ambu^®^ AuraGain^TM^ laryngeal masks, necessitating the use of a smaller tube. While this size difference may not significantly impair air drainage, it could be limiting when dealing with fluids or more solid contents.

Another appealing feature of laryngeal masks is their ability to aid in tracheal intubation in emergency cases [2,3]. In this regard, no significant differences were found among the three devices. A study by Svendsen et al. compared the i-GEL^®^ laryngeal mask to the Ambu^®^ AuraGain^TM^ for facilitating bronchoscopic intubation through the laryngeal mask, finding an 87% success rate with no significant difference between the two [14]. While we did not advance the bronchoscope into the patients’ trachea, the Singularity^TM^ Air had a median Brimacombe score of 4 (clear view to glottis opening) [10], and a median high rating of 4 (potentially very easy; clear sight), as assessed by the responsible investigator.

Finally, among the few adverse events reported, the most common complaints after using all laryngeal masks were sore throat and mild swallowing difficulties or hypo-/dysesthesia. The hematoma resolved spontaneously, and none of the complications persisted. In 2% of the patients, bloodstaining was observed on the tip after removal, a finding somewhat lower than in previous studies [9,12].

It is important to acknowledge some of the limitations of our study design. First, we did not systematically assess the effects of bending the shaft of the Singularity^TM^ Air after insertion. We noted that this laryngeal mask was slightly stiffer and more resistant to insertion. However, once placed, no further adjustments to its positioning were necessary. Second, we did not determine the sample size based on estimates of power needed to detect differences in failure rates among the groups as there is limited data about the Singularity^TM^ Air. Third, we are limited in our ability to draw conclusions about mask performance in difficult airway situations. Fourth, a limitation of the study is its three-arm design, which compared the new device with two established laryngeal masks. Although this design introduced additional complexity, it provided a broader and more clinically relevant evaluation of the new device’s performance against widely used alternatives. Moreover, three-arm comparative designs are not unprecedented in airway device research and have been used in several previous studies to contextualize the performance of new devices against established comparators [20]. Lastly, using real-time randomization software with block randomization or stratification could have helped prevent the unequal allocation of patients across groups. Such a computerized system would have allowed for adjustments in real time, effectively accounting for issues such as canceled operations and ensuring a more balanced distribution of participants.

In conclusion, the outcomes of the recently introduced Singularity Air^TM^ were similar to the widely studied Ambu^®^ AuraGain^TM^ laryngeal mask when used under clinical conditions. Although the i-GEL^®^ laryngeal mask outperformed the Singularity Air^TM^ in certain aspects, such as time to successful ventilation and insertion rate, most of the differences were neither statistically nor clinically significant.

## Figures and Tables

**Table 1:** Demographic and anesthetic characteristics by laryngeal mask type.

	Total Cohort (*n* = 98)	Reference Groups		New Device	*p*-Value ^§^
		Ambu^®^ AuraGain^TM^ (*n* = 29)	i-GEL^®^ (*n* = 40)	Singularity^TM^ Air (*n* = 29)	
ASA physical status * (I–III)	1.9 ± 0.6	2 ± 0.5	1.8 ± 0.6	1.9 ± 0.5	0.1324
Gender (male)	51%	59%	52.5%	41%	0.43
Height (centimeters) *	171 ± 9.8	171 ± 8.6	173.5 ± 10.7	179 ± 9.5	0.3798
Weight (kg) *	77 ± 15	77.9 ± 16	76.7 ± 15	77 ± 14.9	0.9533
BMI (kg/m^2^) *	26 ± 3.7	26.5 ± 3.8	25 ± 3.5	26 ± 3.9	0.3886
Mallampati score (I–III)	1.4 ± 0.55	1.59 ± 0.56	1.31 ± 0.59	1.34 ± 0.54	0.125
Dentures (yes)	4%	3%	7%	--	0.999
Anesthesia duration (minutes) **	75 (59–100)	68 (54–94)	79 (63–105)	69 (59–100)	0.3909

* Mean ± standard deviation; ** median (interquartile range); ASA = American Society of Anesthesiology; BMI = body mass index; ^§^ Bonferroni correction *p* < 0.0167 applied due to three-group comparisons.

**Table 2:** Parameters related to laryngeal mask airway management.

	Total Cohort (*n* = 98)	Reference Groups		New Device	*p*-Value ^§^
		Ambu^®^ AuraGain^TM^ (*n* = 29)	i-GEL^®^ (*n* = 40)	Singularity^TM^ Air (*n* = 29)	
Initial leak pressure (cm H_2_O) *	25 (19–25)	25 (25–25)	22 (15–25)	25 (21–25)	0.0138
Leak pressure after 15 min (cm H_2_O) *	25 (21–25)	25 (25–25)	25 (20–25)	25 (23–25)	0.1148
Insertion first pass rate	65%	52%	88%	48%	0.001
Time-to-first ventilation (seconds) *	33 (26–46)	39 (32–52)	29 (25–42)	34 (29–43)	0.0106
Manual bag-mask ventilation (no intervention)	92%	90%	88%	100%	0.241
Mechanical ventilation (no intervention)	85%	86%	85%	84%	0.807
Gastric tube insertion (no intervention)	84%	93%	70%	93%	0.058
Possible intubation (grading 1–4) *	4 (3–4)	4 (3–4)	4 (4–4)	4 (3–4)	0.4879
Sore throat (NRS 0–10) *	0	0	0	0	0.3248
Hypo-/dysesthesia (yes)	5%	3%	5%	7%	0.859
Blood-stained LM tip (yes)	2%	3%	0%	3%	0.511
Dysphagia (yes)	5%	3%	3%	10%	0.438

* Median (interquartile range); ^§^ Bonferroni correction *p* < 0.0167 applied due to three-group comparisons; LM = laryngeal mask; NRS = Numeric Rating Scale.

## Data Availability

Data supporting the findings of this study can be obtained from the corresponding author upon reasonable request.

## Abbreviations

The following abbreviations are used in this manuscript:

APV	Adaptive pressure release valve
ASA	American Society of Anesthesiologists
BIS	Bispectral index
BMI	Body mass index
CPAP	Continuous positive airway pressure
LM	Laryngeal mask
PEEP	positive end-expiratory pressure
SGA	Supra-glottic airway
TCI	Target-controlled infusion
